# Working relationship between primary and specialist care in analysing elevated liver values—a survey from the point of view of gastroenterologists

**DOI:** 10.1007/s10354-021-00855-5

**Published:** 2021-06-29

**Authors:** Julian Wangler, Stefan Claus, Michael Jansky

**Affiliations:** Research Associate | Centre for General Medicine and Geriatrics, Universitätsmedizin Mainz, Am Pulverturm 13, 55131 Mainz, Germany

**Keywords:** Liver, Transaminases, General practitioners, Algorithm, Early detection, Leber, Transaminasen, Hausarzt, Algorithmus, Früherkennung

## Abstract

Elevated liver values are often an incidental finding in outpatient care. A solid working relationship between general practitioners and specialists plays as much of a role in effective diagnostics as do selection and examination of liver values and context as indicators for referral towards more in-depth diagnosis. This article focuses on the status quo as well as potential hurdles and challenges in the relationship between general practitioners and specialists with regard to analysing elevated liver values of uncertain origin. A total of 529 physicians in gastroenterological practices in the German states of Baden-Württemberg, Hesse and Thuringia were invited to take part in an online survey in 2020, of which 313 responded. This contribution focuses on those parts of the survey covering the relationship between general practitioners and specialists. According to the results, 72% of the surveyed gastroenterologists saw working relationships between general practitioners and specialists as beneficial and effective. Even so, a variety of challenges and difficulties in everyday care dominate. Specialists especially criticised preliminary analyses performed by general practitioners as well as time of referral. Apart from that, a wide majority (85%) saw a major role in a structured diagnostic algorithm towards improving early detection and coordination between primary and specialist care. The survey revealed problems in the relationship between general practitioners and specialists. Together with targeted training and further training programmes for general practitioners, a validated diagnostic algorithm for classifying and analysing elevated liver values may be a valuable tool for general practitioners to perform diagnostics and improve the structure within which they work with specialists.

**Supplementary Information:**

The online version of this article (10.1007/s10354-021-00855-5) contains supplementary material, which is available to authorized users.

## Abstract (de)

In der ambulanten Versorgung treten Leberwerterhöhungen als häufiger Zufallsbefund auf. Für eine effektive Diagnostik ist nicht nur von Bedeutung, welche Leberwerte in welchen Konstellationen als Indikatoren Beachtung finden oder wann Patienten zur weiterführenden Diagnostik überwiesen werden, sondern auch, inwiefern es eine funktionierende Kooperation zwischen Haus- und Facharztebene gibt. Im Mittelpunkt des Beitrags stehen der Status quo sowie mögliche Hürden und Herausforderungen bei der Zusammenarbeit zwischen Haus- und Fachärzten hinsichtlich der Abklärung (unklar) erhöhter Leberwerte. In Baden-Württemberg, Hessen und Thüringen erfolgte im Jahr 2020 eine Online-Befragung von insgesamt 529 Ärztinnen und Ärzten gastroenterologischer Schwerpunktpraxen; letztlich teilgenommen haben 313 Ärztinnen und Ärzte. Der Beitrag fokussiert auf diejenigen Abschnitte der Befragung, die die Kooperation zwischen Haus- und Fachärzten betreffen. Die Ergebnisse zeigen, dass die Zusammenarbeit zwischen Haus- und Facharztebene nach Ansicht der befragten Gastroenterologen in weiten Teilen funktioniert und überwiegend positiv beurteilt wird (72 %). Dennoch ist sie im Versorgungsalltag durch verschiedene Herausforderungen und Schwierigkeiten geprägt. Fachärzte kritisieren insbesondere die im Vorfeld von Hausärzten geleistete Abklärung und Diagnostik sowie den Überweisungszeitpunkt. Zudem erachten sie die Einführung eines strukturierten Diagnosealgorithmus in breiter Mehrheit (85 %) als wichtigen Ansatz für die Verbesserung der Früherkennung und eine bessere Koordination zwischen den Versorgungsebenen. Die Befragung hat Schnittstellenprobleme zwischen Haus- und Fachärzten offengelegt. Ein validierter Diagnosealgorithmus zur Einordnung und Bewertung erhöhter Leberwerte kann in Verbindung mit gezielten hausärztlichen Schulungs- und Fortbildungsformaten ein wertvolles Instrument sein, um Hausärzte effektiv bei der Diagnostik zu unterstützen und die Zusammenarbeit mit Fachärzten besser zu strukturieren.

## Introduction

Around 21,000 people in Germany die every year as a result of liver disease, chronic or otherwise, with liver cirrhosis as the cause in 80% of such cases [1–5]. Long-term increases in liver values play a well-established part in liver-associated mortality in many cases [6–11]. Factors such as alcohol abuse, medication, non-alcoholic fatty liver disease and viral hepatitis also play a role [4, 12–16].

The frequency with which liver values of unknown aetiology are detected as (incidental) findings [17–19] and the role that liver-related disease plays in the healthcare system suggest the importance of timely diagnosis and initiation of targeted treatment. This requires a functioning and sufficiently effective relationship between outpatient primary and specialist care [20]. General practitioners as providers of primary care are often the first to discover moderate increases in liver values in their patients during routine examination [16–19, 21]. This poses general practitioners with the challenge of providing quality differential diagnostic analysis within the constraints of time and cost, and often with only basic resources available to them [21, 22]. This applies to potential warning signs beyond aetiological classification and evaluation of specific elevated liver values [23]. Apart from the question as to which values in which reference ranges and contexts should be included as relevant indicators [19, 22, 24], differentiation plays a crucial role for general practitioners dealing with elevated liver values of unknown aetiology, specifically which cases justify a wait-and-see approach (with repeat laboratory testing) and which cases indicate an immediate need for further diagnostics, for example by direct referral to a specialist or to outpatient liver clinic [19, 21, 22].

Primary medical care has faced criticism for its ineffectiveness in consistent identification and analysis of elevated liver values in German-speaking countries and elsewhere in Europe [5, 15, 17, 24]. This has led to criticism of the low proportion of early diagnoses and lack of standardised differential diagnostic procedures that depend heavily on the respective general practitioner. One reason has been seen as the lack of structured early detection programmes specifically aimed at chronic liver disease as part of standard healthcare [20]. Specifically in Germany, general practitioners currently do not have any evidence-based guidelines appropriate for primary care use.

Gastroenterological specialists receive a significant proportion of their patients with (unclear) elevated liver values by referral from general practitioners. Preliminary assessment from primary care is then either completed, or further examinations are conducted by the specialist [22]. The specialist is responsible for the decision as to whether the patient should be referred back to the general practitioner or to a specialised outpatient clinic as soon as possible. Specialists are also responsible for ensuring proper transfer of findings [19].

German-speaking countries have hardly produced any methodical studies to shed light on the reality of outpatient care in response to elevated liver values of unknown cause. A survey of general practitioners carried out in 2020 demonstrated wide discrepancies in how general practitioners assessed blood analysis findings [25]. Some focused on functional parameters such as bilirubin, blood clotting, cholinesterase and albumin, whereas others looked for indicators of toxic cell damage or manifest liver disease. Most general practitioners were in favour of a wait-and-see approach in response to incidental findings of elevated liver values with uncertain origin.

Problems in the working relationship between primary and specialist care are complex and diverse, especially in analysing elevated liver values. There is a lack of specific findings especially in this respect [22]. The above survey [25] found that general practitioners experienced a variety of challenges with gastroenterological specialists. These challenges related to interdisciplinary communication and transfer of information and findings, behaviour on referral and availability of appointments. Inadequate patient briefing from specialists was also a point of criticism.

So far, no algorithm has been developed to identify patients with elevated liver values as being at high risk of developing liver cirrhosis. This explains why treatment of elevated liver values, especially from blood tests in primary care, has depended so heavily on each individual physician’s approach up to now. International studies suggest that a diagnostic and treatment pathway applied throughout the healthcare field would make the relationship between primary and specialist care more effective while giving differential diagnostic procedures more efficient structure [26–30].

### Research interest

The present work is part of a multistep study that determines the status quo with regard to analysing elevated liver values of unknown aetiology in outpatient care. The primary focus is on general practitioner-based care. The aim of the study is to develop potential approaches towards optimising diagnostics in liver cirrhosis. This contribution focuses specifically on the interdisciplinary relationship between general practitioners and specialists.

The present study has addressed internal and gastroenterological specialists in how they see their relationship with general practitioners in diagnosing and treating patients with moderately elevated liver values, where deficits exist, and which measures would be warranted towards improving early identification of liver disease in outpatient settings.

## Materials and methods

### Study design and survey method

The present study was based on a preliminary study from 2018 [31]. In the preliminary study, a total of 54 gastroenterologists from the states of Rhineland-Palatinate and Saarland were asked how they dealt with elevated liver values in their patients.

The present study interviewed specialists in gastroenterological offices. The original questionnaire [31] was updated and further developed with the aid of literature research. The survey was designed to be exploratory owing to the highly incomplete state of research on how liver disease is currently treated in medical practice. Individual questions—including the wait-and-see approach to elevated liver values and an item battery with suggestions towards early detection—were tightly focused on the primary care survey mentioned at the beginning [25], keeping some of the questions from that survey identical to the questions to be put to the gastroenterologists as far as possible towards facilitating a direct comparison of responses.

Two experts from the Cirrhosis Centre at the Mainz University Medical Centre were consulted while developing the original questionnaire to validate it for completeness and appropriateness from a specialist’s point of view and make any additions necessary. The latter referred to questions on medical office equipment, patient structure and number of diagnosed hepatopathies; these are the only cases not addressed in the following contribution as they have no direct relevance to the relationship between primary and specialist care. The Cirrhosis Centre maintains close contact with a large number of gastroenterological offices, so the Centre’s assistance made it possible to develop the questionnaire closely based on the reality of healthcare and identify relevant issues in the interdisciplinary relationship.

The questionnaire ultimately used focused on a number of questions such as how internists and gastroenterological specialists saw their relationship with general practitioners in treating patients with elevated liver values and the challenges that arose from this relationship. In addition, one item battery in the questionnaire enquired about potentially useful measures to improve early detection of liver disease in primary care. The compact seven-page questionnaire with a completion time of around twelve minutes mostly comprised closed questions with an ‘Other’ option for respondents to give additional information in some cases.

Sociodemographic information included age, gender, federal state in Germany, office setting and type, specialist group, and number of physicians and patients per quarter. The actual regions and districts of the offices were not queried to preserve anonymity and cannot be identified beyond the general information on the office setting. This questionnaire was not subjected to separate pretesting due to the above preliminary questionnaire.

### Recruitment and participants

The anonymised survey was performed between April and October 2020. All 529 specialists in gastroenterological offices in Baden-Württemberg, Hesse and Thuringia were sent written invitations to respond. The cover letter included password-protected access to the online survey. The general practitioners that responded did not receive any financial reward for participation.

### Random sample

A total of 313 completed questionnaires of the 319 questionnaires processed were included in evaluation (59% response rate). The sample was structured as follows:

- Gender: 84% male, 16% female
- Average age: 58 (median: 57)
- Specialist group: 65% specialists in internal medicine and gastroenterology, 28% specialists in internal medicine, 7% other
- Office setting: 67% in medium-sized and large towns or cities, 33% in small towns or rural areas
- Type of office: 40% individual doctor’s offices, 57% joint offices, 3% other
- Patients per quarter: 30% < 1000, 19% 1000–1500, 22% 1500–2000, 29% > 2000

### Ethics

During this study, no sensitive patient data was gathered or clinical tests performed. This is a strictly anonymised survey of a total of 313 gastroenterological specialist practices. However, the authors of the study contacted the Ethics Commission of the State of Rhineland-Palatinate before beginning the study to ensure that it conformed with the medical professional code of conduct.

### Data analysis

After cleansing, we analysed the data using SPSS 23.0 (IBM, Armonk, NY, USA). A factor analysis (Varimax rotation) was carried out. The aim of the factor analysis is to condense a larger number of variables into factors based on systematic relationships (correlations). By condensing the variation of a plurality of variables into a much smaller number of common factors (data reduction), it is hoped to discover underlying common dimensions [32].

In order to check the prerequisite for a factor analysis (Table 1), the Kaiser–Meyer–Olkin sampling adequacy of the random sample was tested first and found to be sufficient in the present case (0.63). Secondly, we carried out Bartlett’s test for sphericity to check the hypothesis that all correlation variables have a value of zero in the basic population. A significant result, as in the present case, allows the interpretation that, in the basic population “there are correlations at least between some variables; the null hypothesis can therefore be rejected” [37, p. 325]. In the case of all included variables, the commonalities are also significantly above the standard threshold 0.5, so that each individual item variable is suitable for the factor analysis. In order to determine the exact number of factors, in addition to considering the Kaiser criterion, the scree test was used. The scree test is a visual test that looks for disjunctures in the pattern of eigen values as a function of factor succession. Values of 0.4/−0.4 were selected as the limits for items to constitute meaningful factors [32].

**Table 1:** Indicators of liver disease onset

		Rotated component matrix		
	Overall agreement [%]	Component 1 (Expl. variation: 28.3%)	Component 2 (Expl. variation: 14.1%)	Component 3 (Expl. variation: 12.2%)
Tiredness and fatigue	93	0.405	0.071	0.837
Years of alcohol consumption	91	0.637	0.183	−0.048
Upper abdominal complaints	84	0.228	0.401	0.721
Suspected alcohol abuse	77	0.420	−0.055	0.485
Characteristic skin alterations (spider naevi etc.)	75	0.689	0.117	0.429
Ascites	73	0.895	0.174	−0.159
Chronic itching	68	0.720	−0.069	−0.180
Loss of appetite	59	0.323	0.804	−0.070
Gynaecomastia	58	0.771	0.062	0.200
Multiple bruises	57	0.742	0.100	0.262
Digestive and bowel issues	55	0.003	0.798	0.299
Changes in weight	54	0.146	0.821	−0.032
Recurrent nosebleeds	47	0.567	0.216	−0.144
Dupuytren’s contractures	38	0.690	−0.261	0.175
Persistent diarrhoea	23	−0.170	0.504	0.332
Genital mycosis	9	0.176	0.162	−0.595
Persistent headache	8	0.066	0.469	−0.131
Carpal tunnel syndrome	5	0.230	0.147	0.062

Question: *In general: What do you see as the most frequent indicators of liver disease onset, and what would prompt you to make more in-depth diagnostics?* (*N* = 313)

Extraction method: Principal component analysis

Rotation method: Varimax, Kaiser normalisation

Rotation in 5 iterations for convergence

Total explained variation: 54.6%

Sampling adequacy, Kaiser–Meyer–Olkin: 0.63

Significance, Bartlett: *p* < 0.001

## Results

### Composition of the patient population

The results confirm the central role of general practitioners in guiding patients through the healthcare system. In response to a question that allowed multiple replies, almost all internal specialists (98%) stated that patients with elevated liver values of unknown aetiology were usually referred by their general practitioners. Of the respondents, 23% responded that another specialist referred patients to their office compared to 20% reporting that their patients presented on the advice of the clinic (40% were direct appointments made by the patient).

### Indicators for in-depth diagnostics

One item battery in the questionnaire asked for frequent indicators of liver disease onset usually leading to more in-depth diagnostics. From previous experience, specialists especially look for excessive alcohol consumption as well as signs such as upper abdominal discomfort, fatigue, ascites, itching and skin alterations (Table 1).

The Varimax factor analysis was chosen to search for underlying common dimensions. As described in the methods section, the statistical requirements for performing the factor analysis were met. The analysis turned out in favour of a three-factor solution, since in the present case three factors have a disproportionately high explanatory power and in each case an eigenvalue > 1 (Kaiser criterion). In addition to this, the explained overall variance is comparatively high (55%) in a three-factor solution. Even according to the scree test, the pattern of eigenvalues most readily points to a three-factor solution. Consequently, such a structure appears to be plausible and stable.

In keeping with the outlined procedure, it is possible to distinguish between three clusters of respondents. The analysis showed different focal points within the sample concerning potential indicators of incipient liver disease. The first and third clusters focused on more common and typical symptoms, whereas physicians in the second cluster also included less common signs of liver damage.

Table 2 shows laboratory values usually examined by specialist respondents. The data show that gastroenterological specialists collect extensive and specific values for more general and special laboratory parameters compared to other settings such as primary care.

**Table 2:** Laboratory values observed

	Overall agreement [%]
γ‑glutamyltransferase (GGT)	100
Aspartate aminotransferase (ASAT, AST, GOT)	98
Alanine aminotransferase (ALAT, ALT, GPT)	97
AP (alkaline phosphatase)	96
Platelet count	85
Bilirubin	84
Ferritin	76
PT according to Quick (INR)	76
Albumin	75
Cholinesterase	75
Hepatitis B/D	75
Hepatitis C	74
MCV	74
Other autoantibodies (ANA etc.)	55
AMA, AMA/M2	49
Anti-LKM, anti-SLA	47
Immunoglobulins	41
Hepatitis E	36
p- and c‑ANCA	32

Question: *Which laboratory values potentially linked to liver disease do you usually examine in routine lab work for general screening check-ups? *(*N* = 313)

Respondents were asked for their opinion on the most important and meaningful indicators for early detection of liver cirrhosis (multiple answers allowed). Like the previous results, responses included γ‑GT (87%), aspartate aminotransferase (82%), alanine aminotransferase (78%), PT according to Quick (61%) and cholinesterase (55%). Other values followed behind by a substantial margin.

Specialists usually had more laboratory budget available to them than general practitioners [19, 27]. This corresponds to the 88% of specialists responding that they frequently collected additional liver-associated laboratory values not determined in advance by the general practitioners, especially AMA, AMA/M2 (88%), ferritin (82%), hepatitis B/D (80%), hepatitis C (79%), and anti-LKM and anti-SLA (78%).

Apart from that, 54% of specialists responded that they often (occasionally in 34%) collected liver-associated laboratory values as controls that had previously been tested in primary care. Note that specialists work with a preselected patient group in interpreting these results.

### Wait-and-see approach

Unlike the many general practitioners that usually prefer the wait-and-see approach towards elevated liver values with unclear aetiology [5, 33], the clear majority of gastroenterological specialists favour referral to a specialist clinic as soon as possible. Only 32% of the doctors surveyed believed in wait-and-see as an effective approach. Note that most patients with elevated liver values presenting to specialists did so on referral from their general practitioners, possibly after a waiting period in primary care. Of the specialists, 76% saw a waiting period of no more than four weeks as appropriate (median: 6.0).

### Steps following diagnosis

Specialists were also asked about the subsequent course taken for patients diagnosed with liver disease onset over the past few years (multiple answers allowed). Of the respondents, 63% stated that these patients remained in their own care for observation or further treatment, 56% reported that they had referred their patients back to their general practitioners for further advice and analysis, and 38% opted to refer them to a specialist liver clinic.

### Working relationship between primary and specialist care

**Table 3:** Challenges experienced in the interdisciplinary relationship

Statement	Frequently [%]	Occasionally [%]
*I have detected (incipient) liver disease that the general practitioner did not notice or remained unaware of in a patient*	25	59
*Primary care could do better at initial testing and diagnosis of (incipient) liver disease*	29	42
*General practitioners are not always sufficiently aware of elevated liver values with unknown aetiology to notice the onset of liver disease at an early stage*	27	43
*Patients that general practitioners have referred to gastroenterologists for elevated liver values of unknown aetiology often turn out to be unspecific*	18	51
*General practitioners often fail to follow up on elevated liver values*	23	42
*General practitioners are too quick to refer patients with elevated liver values of unknown aetiology*	34	30
*General practitioners do not pass on enough information from their own examinations, findings, or diagnoses to gastroenterological specialists*	20	43
*General practitioners are inconsistent in their approach to analysing liver values; this may include varying liver values recorded depending on the general practitioner, so specialists need to keep adjusting to the preliminary work performed by general practitioners*	35	22
*General practitioners are too slow to refer patients with elevated liver values of unknown aetiology*	30	27

Question: *A variety of challenges may arise when gastroenterologists and general practitioners work together to diagnose and treat liver cirrhosis. How often have you experienced the following challenges? *(*N* = 313; only the *Frequently* and *Occasionally* response categories are shown)

As seen in Table 3, gastroenterologists saw the main obstacles in their relationship with general practitioners as being failure to follow up on elevated liver values and referring patients found to have elevated liver values referred to a specialised clinic too soon or too late. Some of the respondents also stated that the vastly different approach taken by general practitioners in analysing elevated liver values such as by collecting deviating liver values represents an additional obstacle, which may play a role in the impression that examinations, results and diagnoses are not always transparent.

Despite these issues in the relationship, 72% of responding specialists were highly or moderately satisfied with their relationships towards general practitioners. Only 25% were moderately or highly dissatisfied (difficult to say or no answer: 3%).

### Approaches to optimisation

In view of the perceived inconsistency in approaches taken towards analysing elevated liver values in outpatient care and the existing issues in the relationship, the responding specialists saw a structured diagnosis and treatment algorithm for general practitioners as especially beneficial in increasing the relative numbers of patients diagnosed at an early stage. Of the respondents, 85% were of the opinion that establishing this treatment pathway would be moderately or highly effective, while 55% consider an extension of the health check-up on patients from the age of 35 to be a moderately or highly effective measure. Furthermore, 52% were in favour of explicit liver checks as part of statutory health insurance, and 76% anticipated that greater numbers of advanced training programmes would support general practitioners and specialists in detecting liver diseases sooner and more effectively.

## Discussion

### Principal findings and comparison with prior work

Proper routine collaboration between general practitioners and specialists undoubtedly plays an essential role in effective, early examination and diagnostic analysis of elevated liver values and treatment specific to the patient [2, 18, 22]. The results presented from the survey amongst gastroenterologists has almost consistently confirmed the findings of the preliminary study [31] and illustrated the generally beneficial and useful working relationship between primary and specialist care in this area. These results also demonstrate specific and divergent points of view in dealing with elevated liver values.

The specialist perspective obtained during the online survey points to a number of challenges in everyday practice that apply to general practitioners and specialists in different ways:

- *Time of referral: *The responding specialists reported that general practitioners often refer patients with elevated liver values of unknown aetiology too early, and these patients yield unspecific results. For comparison, a broad survey of 2700 general practitioners taken in 2020 tallied with these findings: Most general practitioners saw the wait-and-see approach as appropriate, but many referred their patients directly to a specialist in everyday practice [25]. Apart from restrictions due to the healthcare system such as time required for in-depth diagnostics, laboratory budgets and so on, this may be interpreted as an expression of a certain diagnostic uncertainty that may result from the lack of an algorithm with specific guidelines [29].
- *Guidelines: *Amongst other things, specialists criticise the timing with which general practitioners refer patients with elevated liver values. Even so, specialists often also seem to refer the patients back to general practitioners after diagnosing liver disease. Without a rapid referral to a specialist outpatient clinic, the risk emerges of placing patients into an unnecessary referral loop between primary and specialist care. This issue has been raised by general practitioners, who saw the problem exacerbated by specialist findings not always being sent back to them in a timely fashion [18, 19, 22, 33].
- *Liver values and broader context: *Gastroenterological specialists confirmed the results from the survey on general practitioners mentioned in the theoretical part in that general practitioners focus on widely varying liver values in everyday practice [25].
- *Level of knowledge amongst general practitioners:* The responding specialists saw benefit in more training programmes to give general practitioners more assurance in analysing liver values. Other studies have indicated that general practitioners generally take a self-critical approach in self-assessment when it comes to analysing elevated liver values [18, 25].

The difficulties mentioned in the relationship between primary and specialist care may also be a result of the lack of a reliable diagnostic and treatment pathway for general practitioners, especially for patients at high risk of developing liver cirrhosis [4, 22, 26]. This structured diagnosis and treatment pathway applied across the healthcare service may be a valuable tool for professionalising and standardising differential diagnostic procedures; the pathway would improve structures, optimise the flow of information and ease demarcation of responsibilities between primary and specialist care [20, 21, 34]. One remarkable result was that more than 80% of specialists were in favour of establishing a general practitioner algorithm of this type and saw no risk of impingement on their professional authority as specialists. A similar proportion of general practitioners were also found to be in favour of a structured diagnostic algorithm [25].

Various research and support networks as well as specialist associations have long since been campaigning for a methodical diagnostic pathway. This has resulted in algorithms being developed with sufficient application potential for elevated liver values [34–36].

International studies suggest that applying a robust diagnostic algorithm that supports general practitioners and specialists throughout the medical field in detecting, classifying, and assessing elevated liver values may come with decisive improvements such as in cost–benefit, earlier detection, more effective follow-up and treatment tailored to each patient to halt disease progression of cirrhosis and possibly even lead to regression [12, 27–30, 35, 37–39]. This could be combined with additional measures such as targeted further training programmes to cement liver value-associated blood testing in primary care check-ups while also developing well-founded guidelines specifically for general practitioners to identify and deal with elevated liver values [25].

### Limitations and directions for future research

The survey had already undergone conceptual testing in several preliminary studies and adjusted to the outpatient care process. The response rate was high at around 60% compared to all 529 gastroenterologists in the states of Baden-Württemberg, Hesse and Thuringia. Even so, the responses can only be taken as representative to a limited extent. Apart from the limited number of cases, the fact needs to be considered that physicians were recruited in certain regions of Germany. In addition, anonymisation serving as a prerequisite for broad participation prevented any form of tracing respondents back to the parts of the three federal states in which their responses originated. Furthermore, physicians more interested in the subject or more competent took may have taken part in the survey to a greater extent, thus tainting the result. The online survey design may also have resulted in a tendency towards less Internet-aware physicians avoiding participation in the survey.

At this point, the context in which the survey was conducted should also be noted, namely working relationships in dealing with moderately elevated liver values. This is an exploratory study aimed at garnering general opinion. This brief questionnaire cannot be expected to do justice to the complexity of the actual care situation and its patient-specific challenges such as comorbidities and patient age. The limitations to this study also affect content in that alcoholic liver disease is given more emphasis in both patient history and findings at the cost of hepatological issues such as fatty liver, viral liver diseases and systemic autoimmune phenomena, which have been given less attention. When inquiring about interdisciplinary collaboration with general practitioners, items were formulated that describe problematic situations and challenges. This could have led to a stronger criticism from the gastroenterologists surveyed.

## Conclusion

Effective working relationships between primary and specialist care play a crucial role in timely and effective analysis, diagnosis and exclusion diagnostics related to elevated liver values. A sufficiently validated diagnosis and treatment pathway based on the realities of outpatient care may be a valuable instrument in improving existing issues in the relationship while also promoting professionalisation and standardisation of interdisciplinary cooperation. Broader availability of topic-related further training programmes and guidelines developed for general practitioners would appear advisable towards providing more assurance to general practitioners in outpatient care in diagnosing and analysing liver values as well as improving the structure of the relationship between general practitioners and specialists.

## Supplementary Information

Questionnaire (in German)
