# Supplementary material for: Working relationship between primary and specialist care in analysing elevated liver values—a survey from the point of view of gastroenterologists
Source: Wien Med Wochenschr. 2021 Jun 29;171(13-14):301–9. doi: 10.1007/s10354-021-00855-5 (PMC8484199; doi:10.1007/s10354-021-00855-5)
Supplement: Supplementary file 1 — Questionnaire (in German) [file 10354_2021_855_MOESM1_ESM.pdf]

1. Auf welchem Weg kommen Patienten mit (unklar) erhöhten Leberwerten üblicherweise in Ihre Praxis? (Mehrfachangabe möglich)

◯ Überweisung durch den Hausarzt ◯ Überweisung durch einen anderen Facharzt ◯ Auf Anraten der Klinik

◯ Direktes Aufsuchen durch den Patienten ◯ Sonstiges, und zwar: ________________________ ◯ Keine Angabe

2. Wie viele dieser Patienten haben klinische Symptome, wie viele haben lediglich erhöhte Leberwerte?

Klinische Symptome: ◯ Sehr viele ◯ Eher viele ◯ Durchschnittlich ◯ Eher wenige ◯ Sehr wenige ◯ Keine Angabe

Erhöhte Leberwerte: ◯ Sehr viele ◯ Eher viele ◯ Durchschnittlich ◯ Eher wenige ◯ Sehr wenige ◯ Keine Angabe

3. Welche der folgenden Untersuchungen zur Identifizierung und weiteren Abklärung von Lebererkrankungen bieten Sie vor Ort in Ihrer Praxis an?

◯ Oberbauchsonografie ◯ Erweiterte Labor-Diagnostik ◯ Elastografie ◯ Leberbiopsie ◯ Gastroskopie

◯ Ileo-Koloskopie ◯ Sonstiges, und zwar: ___________________________________ ◯ Keine Angabe

4. Allgemein gefragt: Was sind für Sie die häufigsten Hinweise auf eine beginnende Lebererkrankung und veranlassen Sie zu einer tiefergehenden Diagnostik?

◯ Langjähriger Alkoholkonsum	◯ Oberbauchbeschwerden	◯ Stuhlgang- und Verdauungsprobleme
◯ Müdigkeit, Abgeschlagenheit	◯ Rezidivierende Herzbeschwerden	◯ Vermuteter Alkoholmissbrauch
◯ Persistierende Diarrhoe	◯ Chronischer Juckreiz	◯ Rezidivierende Blasenentzündungen
◯ Rezidivierendes Nasenbluten	◯ Anhaltende Kopfschmerzen	◯ Appetitverlust
◯ Gynäkomastie	◯ Ascites	◯ Leberhautzeichen
◯ Karpaltunnelsyndrom	◯ Multiple blaue Flecken	◯ Genitalmykose
◯ Dupuytrensche Kontrakturen	◯ Gewichtsveränderungen	

Weitere : ____

5. Was schätzen Sie: Wie viele Abklärungen von Hepatopathien haben Sie in den zurückliegenden 12 Monaten durchgeführt?

◯ Keine ◯ 1 – 10 ◯ 11 – 20 ◯ 21 – 30 ◯ 31 – 40 ◯ 41 – 50 ◯ 51 – 60 ◯ Mehr als 60 ◯ Keine Angabe

Bitte auf Rückseite fortfahren

6. Bei wie vielen Ihrer Patienten ist in den zurückliegenden 12 Monaten eine Leberzirrhose erstmals diagnostiziert worden?

◯ Keine ◯ 1 – 3 ◯ 4 – 6 ◯ 7 – 10 ◯ 11 – 15 ◯ 16 – 20 ◯ 21 – 25 ◯ Mehr als 25 ◯ Keine Angabe

7. Wie viele Patienten mit Leberzirrhose betreuen Sie in etwa aktuell bei sich in der Praxis?

◯ Keine ◯ 1 – 3 ◯ 4 – 6 ◯ 7 – 10 ◯ 11 – 15 ◯ 16 – 20 ◯ 21 – 25 ◯ Mehr als 25 ◯ Keine Angabe

8. Wie hoch ist der Anteil von Patienten mit Lebererkrankungen in Ihrer Praxis?

◯ Unter 5% ◯ 6 – 10% ◯ 10 – 15% ◯ 16 – 20% ◯ Mehr als 20% ◯ Keine Angabe

9. Welche Laborwerte, die im Zusammenhang mit Lebererkrankungen stehen können, untersuchen Sie üblicherweise bei einer allgemeinen Screening-Untersuchung bei Ihren Patienten?

◯ Alanin-Aminotransferase ◯ Aspartat-Aminotransferase ◯ Gamma-Glutamyltransferase

◯ Cholinesterase ◯ Bilirubin ◯ Albumin ◯ Alkalische Phosphatase

◯ Ferritin ◯ Quick/INR ◯ MCV ◯ Thrombozytenzahl

◯ AMA, AMA/M2 ◯ anti-LKM, anti-SLA ◯ p- und c-ANCA ◯ Weitere Autoantikörper (ANA etc.)

◯ Hepatitis B/D ◯ Hepatitis C ◯ Hepatitis E ◯ Immunglobuline

Weitere : ____

10. Wenn Sie sich festlegen müssten: Was sind aus Ihrer Sicht die <u>drei wichtigsten</u> Indikatoren zur Früherkennung einer Leberzirrhose?

◯ Alanin-Aminotransferase ◯ Aspartat-Aminotransferase ◯ Gamma-Glutamyltransferase

◯ Cholinesterase ◯ Bilirubin ◯ Albumin ◯ Alkalische Phosphatase

◯ Ferritin ◯ Quick/INR ◯ MCV ◯ Thrombozytenzahl

◯ AMA, AMA/M2 ◯ anti-LKM, anti-SLA ◯ p- und c-ANCA ◯ Weitere Autoantikörper (ANA etc.)

◯ Hepatitis B/D ◯ Hepatitis C ◯ Hepatitis E ◯ Immunglobuline

Weitere : ____

11. Wie häufig kommt es vor, dass Sie leberassoziierte Laborwerte zur Kontrolle erheben, die bereits vom Hausarzt ermittelt wurden?

◯ Häufig ◯ Gelegentlich ◯ Selten ◯ Nie ◯ Keine Angabe

12. Wie häufig kommt es vor, dass Sie zusätzliche leberassoziierte Laborwerte erheben, die nicht vom Hausarzt ermittelt wurden?

◯ Häufig ◯ Gelegentlich ◯ Selten ◯ Nie -> Bitte direkt zu Frage 14 übergehen ◯ Keine Angabe

13. Um welche zusätzlich erhobenen Werte handelt es sich?

◯ Alanin-Aminotransferase ◯ Aspartat-Aminotransferase ◯ Gamma-Glutamyltransferase

◯ Cholinesterase ◯ Bilirubin ◯ Albumin ◯ Alkalische Phosphatase

◯ Ferritin ◯ Quick/INR ◯ MCV ◯ Thrombozytenzahl

◯ AMA, AMA/M2 ◯ anti-LKM, anti-SLA ◯ p- und c-ANCA ◯ Weitere Autoantikörper (ANA etc.)

◯ Hepatitis B/D ◯ Hepatitis C ◯ Hepatitis E ◯ Immunglobuline

Weitere : ____

14. Über den Umgang mit moderat erhöhten Leberwerten gibt es unter Ärzten unterschiedliche Meinungen. Die einen halten es für besser, nach der Feststellung moderat erhöhter Leberwerte erst einmal abzuwarten und zu einem späteren Zeitpunkt eine erneute Untersuchung der Leberwerte durchzuführen. Die anderen bevorzugen eine möglichst direkte Überweisung zum Facharzt bzw. an eine Spezialambulanz. Einmal von speziellen oder eindeutigen Einzelfällen abgesehen, was halten Sie grundsätzlich für sinnvoller?

◯ Kontrolliertes Zuwarten ◯ Überweisung an Facharzt ◯ Überweisung an eine Leberspezialambulanz

◯ Schwer zu sagen, Unentschieden ◯ Keine Angabe

15. Was ist Ihrer Ansicht nach ein adäquater Zeitraum für ein kontrolliertes Zuwarten?

________________________ Wochen

Bitte auf Rückseite fortfahren

16. Wenn Sie an die Patienten denken, bei denen Sie in den letzten Jahren eine beginnende Lebererkrankung festgestellt haben: Worin bestand für Sie im Anschluss an die Diagnose der nächste Schritt?

◯ Patient bleibt zur Beobachtung bzw. Weiterbehandlung in meiner Sprechstunde ◯ Überweisung an eine Leberspezialambulanz ◯ Rücküberweisung zum Hausarzt zwecks weiterer Beratung bzw. Abklärung

◯ Sonstiges, und zwar: ___________________________________ ◯ Keine Angabe

17. Wie häufig kommt es vor, dass Sie bei Patienten eine (beginnende) Lebererkrankung entdeckt haben, auf die der Hausarzt nicht aufmerksam wurde bzw. die beim Hausarzt unerkannt blieb?

◯ Häufig ◯ Gelegentlich ◯ Selten ◯ Nie

18. Was würden Sie sagen: Wie viele Patienten, die mit erhöhten Leberwerten an Ihre Praxis überwiesen werden, erweisen sich letztlich als unspezifisch?

◯ Sehr viele ◯ Eher viele ◯ Durchschnittlich ◯ Eher wenige ◯ Sehr wenige ◯ Schwer zu sagen

19. Wie beurteilen Sie aus Ihrer eigenen Erfahrung die Zusammenarbeit zwischen niedergelassenen Gastroenterologen und Hausärzten, wenn es um die Abklärung unklarer Leberwerterhöhungen oder der Diagnosestellung Leberzirrhose geht?

◯ Sehr gut ◯ Eher gut ◯ Eher schlecht ◯ Sehr schlecht ◯ Schwer zu sagen ◯ Keine Angabe

20. Und wie beurteilen Sie aus Ihrer eigenen Erfahrung die Zusammenarbeit zwischen niedergelassenen Gastroenterologen und Leberspezialambulanzen, wenn es um die Abklärung unklarer Leberwerterhöhungen oder der Diagnosestellung Leberzirrhose geht?

◯ Sehr gut ◯ Eher gut ◯ Eher schlecht ◯ Sehr schlecht ◯ Schwer zu sagen ◯ Keine Angabe

21. Bei der Zusammenarbeit zwischen niedergelassenen Gastroenterologen und Hausärzten zur Abklärung einer Leberzirrhose können verschiedene Schwierigkeiten auftreten. Wie häufig treten die folgenden Schwierigkeiten Ihrer Erfahrung nach auf?

Hausärzte leiten Patienten vorschnell zum Gastroenterologen weiter, wodurch gastroenterologische Gebietsärzte auf lange Zeit ausgebucht sind.

◯ Häufig ◯ Gelegentlich ◯ Selten ◯ Nie

Hausärzte informieren gastroenterologische Gebietsärzte nicht ausreichend über die von ihnen durchgeführten Untersuchungen, Ergebnisse und/oder gestellte Diagnosen.

◯ Häufig ◯ Gelegentlich ◯ Selten ◯ Nie

Der Wissensstand von Hausärzten im Zusammenhang mit (unklar) erhöhten Leberwerten ist nicht immer ausreichend, um frühzeitig auf eine beginnende Lebererkrankung aufmerksam zu werden.

◯ Häufig ◯ Gelegentlich ◯ Selten ◯ Nie

Die von Hausärzten zum Gastroenterologen weitergeleiteten Patienten mit (unklar) erhöhten Leberwerten erweisen sich häufiger mal als unspezifisch.

◯ Häufig ◯ Gelegentlich ◯ Selten ◯ Nie

Hausärzte warten zu lange, bis sie Patienten mit (unklar) erhöhten Leberwerten beim Gastroenterologen vorstellen.

◯ Häufig ◯ Gelegentlich ◯ Selten ◯ Nie

Die von Hausärzten im Vorfeld geleistete Abklärung bzw. Basisdiagnostik zur Erkennung von (beginnenden) Lebererkrankungen könnte besser sein.

◯ Häufig ◯ Gelegentlich ◯ Selten ◯ Nie

Viele Hausärzte überweisen direkt zu einer Leberspezialambulanz, obwohl der niedergelassene Gastroenterologe der bessere Ansprechpartner wäre.

◯ Häufig ◯ Gelegentlich ◯ Selten ◯ Nie

Leberwerterhöhungen werden von Hausärzten oft nicht weiter verfolgt.

◯ Häufig ◯ Gelegentlich ◯ Selten ◯ Nie

Der Zeitdruck in der gastroenterologischen Praxis ist so groß, dass zwecks weiterer Abklärung zum Hausarzt zurücküberwiesen werden muss.

◯ Häufig ◯ Gelegentlich ◯ Selten ◯ Nie

Hausärzte meiden die Abklärung von Leberwerterhöhungen, weil die Vergütung im Verhältnis zum Aufwand nicht attraktiv genug ist.

◯ Häufig ◯ Gelegentlich ◯ Selten ◯ Nie

Die fachärztliche Versorgungsdichte in meiner Region ist nicht ausreichend, sodass viele hausärztliche Patienten mit Leberwerterhöhungen nicht adäquat und zeitnah versorgt werden können.

◯ Häufig ◯ Gelegentlich ◯ Selten ◯ Nie

Bitte auf Rückseite fortfahren

22. Aufgrund der zunächst relativ unspezifischen Symptomatik wird die Diagnose einer Leberzirrhose erst in fortgeschrittenem Stadium gestellt. Zur Verbesserung der Früherkennung gibt es den Vorschlag, einen strukturierten Diagnose- und Therapiealgorithmus zu etablieren, der Hausärzten bestimmte Verhaltensanweisungen zum Umgang mit erhöhten Leberwerten gibt und die Versorgungskoordination mit gastroenterologischen Fachärzten verbessert.

Für wie effektiv halten Sie eine solche Maßnahme, um den Frühdiagnose-Anteil von Patienten zu erhöhen?

◯ Sehr effektiv ◯ Eher effektiv ◯ Weniger bzw. gar nicht effektiv ◯ Weiß nicht

23. Wie kompetent fühlen Sie sich, wenn es darum geht, den Verdacht auf eine Lebererkrankung nach Feststellung erhöhter Leberwerte abzuklären?

◯ Sehr kompetent ◯ Eher kompetent ◯ Eher nicht so kompetent ◯ Keine Angabe

24. Wie kompetent fühlen Sie sich bei der Behandlung und Betreuung von Patienten mit Leberzirrhose?

◯ Sehr kompetent ◯ Eher kompetent ◯ Eher nicht so kompetent ◯ Keine Angabe

25. Wie würden Sie das generell einschätzen: Sind die meisten fachärztlich tätigen Internisten und Gastroenterologen ausreichend kompetent, wenn es darum geht, Lebererkrankungen abzuklären, oder sehen Sie hier Nachholbedarf?

◯ Ausreichend kompetent ◯ Gewisser Nachholbedarf ◯ Großer Nachholbedarf ◯ Schwer zu sagen

Nun bräuchten wir noch einige statistische Angaben von Ihnen. Wie auch der Rest des Fragebogens werden diese Informationen natürlich streng vertraulich behandelt und anonymisiert.

Geschlecht: ◯ Männlich ◯ Weiblich

Ihr **Alter**: _____

Ihr **Bundesland**: ◯ Baden-Württemberg ◯ Hessen

Wo befindet sich Ihre Praxis? In einer Gemeinde/Stadt mit . . .

◯ mehr als 100.000 ◯ 20.000-100.000 ◯ 5.000 bis 20.000 ◯ unter 5.000 Einwohnern

Welches Niederlassungsmodell beschreibt Ihre Praxis am besten?

◯ Einzelpraxis (Praxisinhaber ist der einzige Arzt) ◯ Einzelpraxis mit angestellten Ärzten * ◯ Gemeinschaftspraxis *

◯ Medizinisches Versorgungszentrum *

Sie sind…

◯ Facharzt für Innere Medizin ◯ Facharzt für Innere Medizin und Gastroenterologie

◯ Sonstiges, und zwar: ____________________________________

*** Wie viele Ärzte** sind in Ihrer Praxis tätig?

◯ 1 Arzt ◯ 2 Ärzte ◯ 3 Ärzte ◯ Mehr als drei Ärzte

Wie viele Patienten behandelt Ihre Praxis im Quartal?

◯ 750-1000 ◯ 1000-1500 ◯ 1500-2000 ◯ Mehr als 2000

<u>Vielen Dank für Ihre Teilnahme!</u>

Was möchten Sie uns noch mitteilen?
Hier finden Sie Platz für Anregungen, Kommentare und Kritik.

____

____

____
